# Applications of Carbon Nanotubes in the Internet of Things Era

**DOI:** 10.1007/s40820-021-00721-4

**Published:** 2021-09-11

**Authors:** Jinbo Pang, Alicja Bachmatiuk, Feng Yang, Hong Liu, Weijia Zhou, Mark H. Rümmeli, Gianaurelio Cuniberti

**Affiliations:** 1 Collaborative Innovation Center of Technology and Equipment for Biological Diagnosis and Therapy, Institute for Advanced Interdisciplinary Research (iAIR), Universities of Shandong, University of Jinan, Shandong, Jinan 250022 People’s Republic of China; 2 PORT Polish Center for Technology Development, Łukasiewicz Research Network, Ul. Stabłowicka 147, 54-066 Wrocław, Poland; 3 Centre of Polymer and Carbon Materials, Polish Academy of Sciences, M. Curie‐Sklodowskiej 34, 41-819 Zabrze, Poland; 4 Department of Chemistry, Southern University of Science and Technology, Shenzhen, 518055 People’s Republic of China; 5 State Key Laboratory of Crystal Materials, Center of Bio & Micro/Nano Functional Materials, Shandong University, 27 Shandanan Road, Jinan, 250100 People’s Republic of China; 6 College of Energy, Institute for Energy and Materials Innovations, Soochow University, Suzhou, Soochow, 215006 People’s Republic of China; 7 Key Laboratory of Advanced Carbon Materials and Wearable Energy Technologies of Jiangsu Province, Soochow University, Suzhou, 215006 People’s Republic of China; 8 Centre of Polymer and Carbon Materials, Polish Academy of Sciences, M. Curie Sklodowskiej 34, 41-819 Zabrze, Poland; 9 Institute for Complex Materials, Leibniz Institute for Solid State and Materials Research Dresden (IFW Dresden), 20 Helmholtz Strasse, 01069 Dresden, Germany; 10 Institute of Environmental Technology, VŠB-Technical University of Ostrava, 17. Listopadu 15, Ostrava, 708 33 Czech Republic; 11 Institute for Materials Science and Max Bergmann Center of Biomaterials, Center for Advancing Electronics Dresden, Technische Universität Dresden, 01069 Dresden, Germany; 12 Dresden Center for Computational Materials Science, Dresden Center for Intelligent Materials (GCL DCIM), Technische Universität Dresden, 01062 Dresden, Germany

**Keywords:** Carbon nanotubes, Transistors, Sensors, Actuators, Brain–machine interfaces, Energy storage

## Abstract

The post-Moore's era has boosted the progress in carbon nanotube-based transistors. Indeed, the 5G communication and cloud computing stimulate the research in applications of carbon nanotubes in electronic devices. In this perspective, we deliver the readers with the latest trends in carbon nanotube research, including high-frequency transistors, biomedical sensors and actuators, brain–machine interfaces, and flexible logic devices and energy storages. Future opportunities are given for calling on scientists and engineers into the emerging topics.

## Highlights

- The Internet of Things era related electronics were updated based on carbon nanotube transistors, radiofrequency circuits and energy storage devices.
- The applications in healthcare and biomedical devices were discussed including sensory, data processors and actuators.
- The fabrication of wafer-scale carbon nanotubes has been introduced as well as the machine learning strategy for prediction of optimal synthesis parameters.

## Graphical abstract

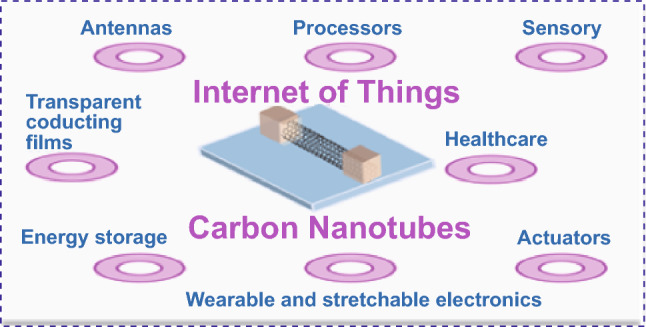

## Introduction

The integration of more transistors in a chip has facilitated improved circuit performances for meeting the requirement of Internet of Things [1–4], which feature the emerging trend of 5G communication, cloud computing, and lightweight consumer electronics. Indeed, the currently available transistors for high-frequency electronics rely on three types of materials, i.e., Si-based complementary metal oxide semiconductor, GaAs and carbon nanotubes. The former two types of materials do not meet the strict requirement of radio-frequency transistors. Therefore, the carbon nanotube-based transistors have provided an effective solution for the post-Moore's era. In this perspective, we list the applications of carbon nanotubes in emerging electronics, such as high-frequency transistors and the Internet of Things [5]. Also, the biomedical engineering of carbon nanotubes is demonstrated by the brain–machine interface and actuators for artificial muscles. Next, the trends for materials optimization and properties prediction are given based on big data and machine learning approaches. Eventually, future opportunities for carbon nanotubes research are delivered to the readers.

## Emerging Opportunities for Carbon Nanotube Applications

### The Internet of Things (IoT)

**Fig. 1:**
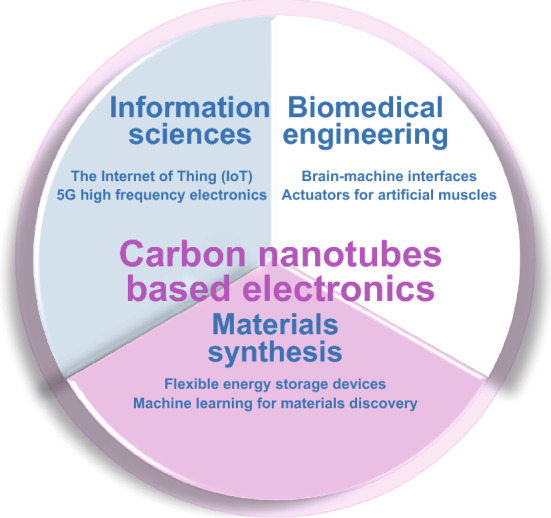
The emerging applications of carbon nanotube-based electronics

As a computing ecosystem, IoT connects everything with embedded electronics through wireless communication. In the IoT system (Fig. 1), sensors first acquire the physical and environmental variables, process the electrical signals, and upload the information wirelessly to a processor for computing [6].

Carbon nanotubes have been aligned with shear forces and deposited as thin film onto dielectric/metal substrate for fabricating microstrip patch antennas [7]. The weight saving of CNT antenna for radio-frequency communication has achieved 5% compared with copper antenna [8]. The CNT-based antennas can be integrated into flexible and wearable devices for information transmission and reception. Carbon nanotubes-based antennas show high radiation efficiency at 10 GHz, comparable with copper antennas [8].

The random-access memory based on carbon nanotubes has been proposed for boosting the reading/writing rate by processor [9–11]. Besides, the composite materials have been developed for the non-volatile memory [12] for data storage [13]. The input devices start emerging with the keypad [14], joystick [15], and touchpad [16]. Meanwhile, the output devices such as display were demonstrated based on CNT driving electrodes [17] and lightening [18–20]. There emerge the carbon nanotube-based analog circuits [21]. Besides, the terahertz imaging system based on CNT has promised the non-destructive detection of industrial products [22].

Hardware true random number generators (TRNGs) are utilized to generate encryption keys for allowing access to sensitive data. The Internet of Things requires flexible true random number generators. The TRNG of SWCNTs [23] has been demonstrated with the devices of static random-access memory. The security protocols are strictly robust, with random numbers from digitizing thermal noise into random 0 and 1 numbers.

### Transparent Conducting Films

The performances of CNT-based transparent conducting films were comparable to the ITO films a decade ago [24, 25]. It has been demonstrated in the touch panel for smart phone [26] and other transparent devices [27–29]. Then, the transparent films were printed with the inks of CNTs [30] or mixtures [31, 32]. The doping of Au nanoparticles could enhance the conductivity of CNT film [33]. Recently, the CNT-based transparent conducing film has been fabricated with blown aerosol technique assisted CVD [34], which demonstrated a high optoelectronic performance, i.e., 90% transmittance and 40 Ω sq^−1^ sheet resistance. The upcoming opportunities remain in the roll-to-roll technique, blending of carbon nanotubes with the metal nanomesh for both improving the transmittance and conductivity as well as reducing the fabrication cost. In addition, the heat dissipation [35] could be a bonus when integrating CNTs into the smart phones.

### Wearable and Stretchable Electronics

Wearable and stretchable electronic devices are often fabricated onto polymer materials [36, 37] or fabric [38, 39], which could be produced by fiber-to-yarn conversion compatible with textile manufacturing [40, 41]. The devices have advantages of stretchability for the production of sportswear [42], nano-energy generation [43], and implantable healthcare devices [44]. Due to the extraordinary mechanical and electronic properties of CNTs, they have been separated [45] and blended [46] into the polymer-based composite yarns for strain sensing [47], triboelectric energy production [48], and health monitoring [49]. Besides, the flexible CNT-based integrated circuits demonstrate the advantage of low energy consumption [50]. Future opportunities remain in the mechanical durability of materials and retention of device performances for long-term operation and wearing.

### Mimicking Human Sensory Systems

**Fig. 2:**
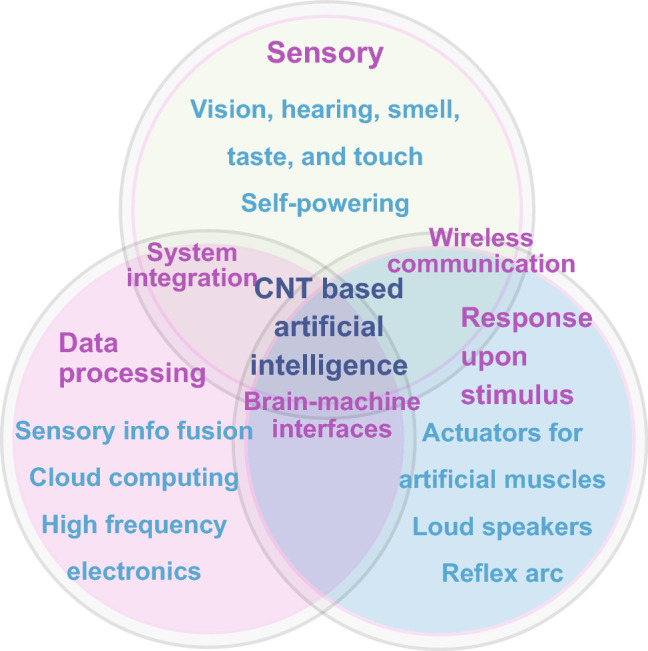
The system integration of carbon nanotubes-based artificial intelligence. Three kinds of devices compose the system, including the Internet of Things-based sensory, data processing, and response

The five conventional human sensory are vision, hearing, smell, taste, and touch [51–53]. With the processing and comprehension of these five types of sensing signals, the brain assists the human beings to understand the world and generate reflexes upon stimulus [54, 55]. Here, the update of the carbon nanotube-based sensors for mimicking the five sensory are briefly listed as follows (Fig. 2). First, CNT-based retina converts the projected image, i.e., illuminating light, into electrical pulses [56], which imitates the photodetection [57] and guarantees machine vision [58]. Second, the eardrum was fabricated of CNT piezoresistance devices [59]. Third, the electronic nose detects the flavors with a chemoresistive sensor array [60, 61]. Forth, the electronic tongue can recognize the taste by the detection of liquid substances. Typically, electrochemical devices were employed for sensing the glucose [62] and tea taste [63]. Fifth, the tactile sensors lead to the development of electronic skins [64–66]. The fusion of these five sensory will generate a precise acquisition of the environmental information by the smart sensor systems.

### Healthcare Products

The CNT-based devices show great promises in healthcare and biomedical devices. The CNT materials have shown efficient therapy in musculoskeletal tumors [67], restoration of neural activity [68], engineering vascularized oriented tissues [69], and treatment of cancer [70, 71] as well as artificial joint materials [72]. Besides, CNT transistor-based sensors provide early diagnosis by acquiring the sodium concentration of sweat [73] and respiration gases [74]. The electronic skin based on piezoelectronics and synaptic transistor [75] provides promising intelligent prosthetics [76]. The strain engineering of CNTs leads to the health monitoring, i.e., the recognition of human motion [77, 78], the collection of arterial pulse waves [79], and electrocardiogram signals [80]. Future attention could be paid onto the implantable and biodegradable devices for medical curing, data processing [81] as well as multiple sensing platform for real-time health monitoring.

### Actuators for Artificial Muscles

Carbon nanotubes sheets have been testified as electromechanical actuator for generating large stress and strain at several volts [82]. The CNTs, as actuation electrodes [83], were integrated and packaged into a nanofiber yarn, which formed an electrochemical cell by stacking the separator, electrodes, and electrolytes. Moreover, micrometer-scale robots [84] have been reported with electrochemical actuators driven by the voltage from silicon photovoltaic devices. It provides a universal platform [85] for incorporating half a century of knowledge in electronics techniques. Electromechanical SWCNT actuators have shown excellent performances with high stress and strain with the mechanism of double-layer charging [82].

Moreover, carbon nanotube aerogel has mimicked the function of artificial muscles and bionic soft robots in object motion. Yarns of graphene/CNT exhibit the role of artificial muscles [86]. Besides, the elastomer/CNT composite demonstrates high deformation capability upon photothermal stimuli [87]. Then, the elastomer/CNT composite renders an actuator for the shaping and locomotion of soft robotics [88].

### Brain–Machine Interfaces

Neural interfaces [89] have been designed for direct communication with neural tissues. The CNT fiber has rendered an electrode for magnetic resonance imaging (MRI) examination [90]. Compared with commercial Pt/Ir electrode, the CNT fiber as a brain–machine interface [91] decreases its diameter to 5 nm with advantages of easy repositioning and long duration detection. Moreover, CNT fiber has been employed in the recordings and stimulations of neuronal electrical activity [92]. Close electrode-tissue contact with excellent electrical fidelity has been created with a composite of carbon nanotubes and poly(3,4-ethylenedioxythiophene) (PEDOT) as a thin interface layer [93]. The electrochemical impedance of such an electrode exhibits a 50 times reduction compared with a pure gold electrode with the stimulation of biological 1 kHz signal. Besides, a similar composite of CNT/DNA/silica provides an intimate interface for stem cell cultivation [94].

In addition, CNT/polyethylene terephthalate (PET) as a tape [95] has led to imaging and circuit analysis of brain ultrastructure analysis. Besides, the stretchable ionics based on flexible hydrogels show promising applications in the human–machine interface [96].

### Flexible Energy Storage Devices

Wearable electronics and the Internet of Things demand flexible and stretchable energy storage devices such as micro-supercapacitors [115, 116] and thin-film secondary ion batteries [117]. Indeed, commercial lithium-ion batteries and supercapacitors are generally rigid and heavyweight. Therefore, stretchable energy storage emerges for satisfying integrated miniaturized energy storage [118] for consumer electronics [119]. This section briefly lists the recent advances for flexible energy storage devices, including materials innovation [120] and architecture development [121, 122].

One focuses on the anode materials when developing conventional lithium-ion batteries [123]. Indeed, various anode materials are based on carbon nanotubes. They are different composites such as yarns of carbon nanotubes and their fiber composite [118].

But for flexible lithium-ion batteries [124], the complete battery architecture shall be compatible with flexibility and stretchability. Indeed, thin-film lithium-ion batteries with solid electrolytes retain a large energy density. For example, porous textile conductor [125], as an alternative of metal collector, has shown high mass loading of anode materials and large capacity with flexibility. Moreover, CNT films have rendered the binder-free and current collector-free anode materials for the flexible lithium-ion batteries [126]. The efforts for fabricating low-cost secondary ion batteries are made in the intercalation and extraction of larger ions other than lithium ions [127], e.g., zinc [128], sodium [129], and potassium [130].

In addition, flexible CNT-based biofuel cells demonstrate a high-power density, which is conformal as integrated into a cotton textile cloth [131]. The fiber modified by enzyme/carbon nanotube composite guarantees the power supply when bending into an S shape. Besides, carbon nanotubes serve as metal-free catalyst [132], e.g., for flexible Li-CO_2_ batteries [133].

Micro-supercapacitors serve as the flexible power sources with the advantages of long lifetime and high-power density. Different CNT/polymer composites have been developed for high-performance flexible supercapacitors [134]. The CNT/poly(3-methylthiophene) composite provides high pseudocapacitance in an asymmetric supercapacitor [135]. Yarn of CNTs shows superior electrode performances in supercapacitors for textiles [136]. CNTs provide a large specific area for depositing MnO_2_ of pseudocapacitance [137]. The supercapacitors show excellent gravimetric capacitance with electrodes of CVD grown helically coiled carbon nanotubes over carbon fiber. The CNT-based hybrid hydrogel demonstrates environmentally friendly electrode material with dissolving salt in water as an electrolyte for supercapacitor [138]. And the CNT aerogels provide high-rate capacitive performances [139].

Future opportunities in flexible supercapacitors emerge with continuously improving the areal and volumetric capacitance [140], large specific area [141], the handing rate, and the interfaces for coupling various energy nanogenerators. Besides, the CNT-based inks could facilitate the direct printing of supercapacitor electrodes [142]. The understanding of storage mechanisms matters for promoting the performances [143].

General requirements remain for both batteries [144] and supercapacitors, i.e*.*, the lightweight [145], facile synthesis strategies [146], mass production, and mechanical stability [147]. Besides, the stretchability and conformal adhesion with textiles matters, i.e*.*, CNT fibers could be woven into textiles as conducting electrodes for supercapacitors and batteries [148]. The continuous advances of CNT composites-based electrodes [149, 150] require the understanding of the performance enhancement mechanism by synergistic effect [151]. Besides, stretchable solid-state electrolytes are still required to match the available architecture of supercapacitor and batteries, such as cross-linked gel electrolyte or human sweat on carbon thread [152]. In addition, soft packing materials still recall efforts for their optimization and developments, such as human-skin comfortable materials [153] and self-healing polymers [154, 155]. Eventually, the safety and production costs are topmost for paving the way for practical products [156].

Besides, carbon nanotubes have shown the high capability of storing mechanical energy [157], e.g*.*, flywheels for kinetic energy storage [158], which could be utilized in an uninterruptable power supply. CNT yarn twist can convert mechanical energy into electricity [159].

### System Integration

In consumer electronics, the applied electronics as a system require the integration of multiple functional devices, including sensing and signal processing, data communication, and data display. In the Internet of Things era, the wireless sensing becomes dominant. Here, CNT-based electronic systems employ the wireless communication modules including the Bluetooth communication [97], and data acquisition with WiFi route [98], RFID-based wireless data-transmitting sensor [99]. Besides, the human–machine interaction provides the approaches of obtaining human gesture and motion signals [100]. In addition, CNT devices guarantee the remotely controlled actuation [101], and wireless energy transfer [102]. The upcoming efforts should be put into the self-powered sensing system by energy harvesting from the environment and motion energy.

## Materials Optimization Based on Machine Learning

**Fig. 3:**
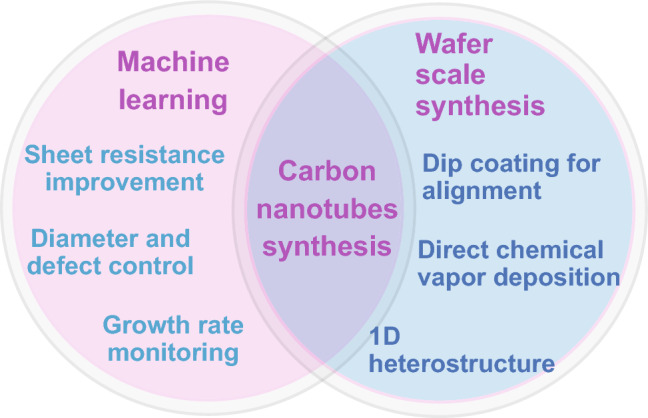
The upcoming machine learning algorithms for obtaining the properties, quality, and growth rate of carbon nanotube synthesis as well as the target of wafer-scale carbon nanotube synthesis

The CNT synthesis has evolved continuously with the assistance of machine learning as well as the wafer-scale preparation (**Fig. ****3**).

*Machine learning for materials discovery.* The quantum computational chemistry has boosted the materials science by providing structure–property relation [103]. With the aid of machine learning [104], the computational chemistry shows the capability of predication of the composition, structure, and properties of existing and unknown materials. Indeed, machine learning provides the design rule and guidelines for new materials findings [105–107].

Furthermore, the chemical reaction processes could be calculated with machine learning [108, 109]. Two mainstreaming algorithms, i.e., support vector regression [110] and artificial neural networks [111], are being developed for optimizing the chemical processes, including the catalysis [112] and carbon nanotube growth [113].

Firstly, the machine learning based on support vector regression algorithm [110] leads to the direct generation of multiple parameters of the optimal synthesis conditions, which is superior to human-centered parameter optimization, viz., one can only optimize one individual parameter (other than several parameters) in the same synthesis operation. By such a set of optimal parameters, the synthetic carbon nanotubes show improved sheet resistance [110] by the floating-catalyst chemical vapor deposition.

Secondly, an artificial neural network algorithm-based machine learning has been utilized for the guide of experimental parameters over the synesthetic CNT quality [111]. Indeed, five synthesis parameters, e.g., the pressure of feedstock, types of feedstocks, substrate temperature, and synthesis time, have been chosen as input for the machine learning. The calculated output data predict the quality of the synthetic CNTs, i.e., yield, tube diameter, and defects, which matches the characterization of experimentally synthetic CNTs well.

Third, the autonomy for material design and performance predictions has been developed by assembling a research robot, termed Autonomous Research System, with machine learning and artificial intelligence techniques [114]. Here, the growth rate of CNTs can be extracted by automated instrument operation for hundreds of experiments, with the assistance of in situ Raman characterization as closed-loop feedback. Indeed, such a robot based on machine learning may accelerate the materials discovery by reducing the participation of human resources and other production cost.

Wafer-scale deposition of aligned CNTs is highly preferred for the fabrications of transistor-based device arrays. Two mainstreaming routes remain for continuous evolution. One is the solution processing-based strategy [165, 166], which involves the dip-coating [167, 168] or vacuum filtration [169] of semiconducting nanotubes, after going through CVD production, dispersion, centrifugation, and sorting. At early stage, CNT thin-film transistors have been developed for integrated circuits [169] and artificial skins [170]. The CNT dispersions serve as inks, which are highly compatible with printed electronics [171]. Based on this approach, functional devices based on individual carbon nanotube transistors have been fabricated for arithmetic logic unit [172], ring oscillators [173], analog amplifiers [174], and DNA recognition [175] applications. Also, the CNT transistors can be fabricated onto flexible biodegradable surfaces [176]. Besides, the carbon nanotube-based heterostructures have shown success in logic inverters [177], photodetectors [178], and solar cells [179]. The second route, termed dry processing [180], can be divided into two categories, i.e., direct CVD growth of horizontal CNTs over dielectric substrates [181] and stretching-pressing of CNT vertical forest film [182]. The horizontally aligned CNTs are preferable for individual CNT transistors [183] while the CNT films are good for thin-film transistors or conductors for touch screen and displays. The horizontally aligned CNTs have led to the iontronics and biocomputing [184]. Future efforts are still required for reducing the production cost and improving the compatibility with the Si-based processing techniques.

## Perspective and Summary

The carbon nanotubes have been intensively investigated for near three decades, but controlled growth of SWCNTs with specific structure and properties remain still challenging. Recent progress on growing specific chirality SWCNTs indicates that catalyst design and growth kinetics are two key points. However, the mechanism of chirality-controlled growth is still unclear. Thanks to the recently developed advanced *in situ* techniques [160, 161], such as aberration-corrected environmental TEM and X-ray absorption, atomic scaled and dynamic information on catalyst and nanotube have been achieved [162]. However, the relation between CVD condition depended SWCNT growth kinetics is complicated to reveal with an *in situ* means, which bring more complex mechanisms [163]. More chiral SWCNTs with high purity need to be achieved by the precise catalyst design and modulation of growth conditions [164]. The cloning growth of SWCNTs from their segments is promising; however, the improvement of growth efficiency and chiral selectivity remain two challenges. Indeed, the control in synthesizing the specific chirality still requires excellent input from the community. Besides, the controlled CNT-based heterostructures become emerging trends for compatibility with device configurations.

In individual theoretical work, the entropy in thermodynamics has driven the formation of chirality-specific carbon nanotubes [185], which may enrich the big data of synthesis parameters and resultant features of carbon nanotubes. Therefore, big-data-driven research could accelerate the materials discovery and feedback the hardware for operating machine learning [186].

The physical and chemical properties of carbon nanotubes remain hot topics. First, the mechanical properties of individual chiral single-walled carbon nanotube are still of great interest, i.e*.*, superlong fatigue lifetime [187]. Indeed, the noncontact acoustic resonance examination enables the *in situ* fatigue tests. Besides, high tensile strength beyond 80 GPa has been achieved with bundles of carbon nanotubes [188].

Breakthrough has been made on carbon nanotube-based electronics, e.g*.*, carbon nanotube transistors, transparent conducting films, triboelectric nanogenerators, and electronic skins. Quite recently, the alignment of dense semiconducting carbon nanotubes has been reported with transistor performances [189], exceeding silicon techniques based on conventional metal–oxide–semiconductor configurations. The high integration density of CNT transistors with wafer-scale homogeneity may demonstrate superior to conventional silicon electronics. Recently, a 16-bit microprocessor has been fabricated with 14,000 CMOS CNT transistors [190]. Furthermore, the three-dimensional integration has emerged with incorporating the complete units of von Neumann architecture into one single chip [191], i.e*.*, the central processor of CNT FET-based logic circuits, data storage with resistive random-access memory, input, and output. The device physics of individual single-walled carbon nanotube requires experimentally proven progress in theoretical predictions.

The development of memristors [192] and ionic floating-gate transistor arrays [193] have shed light on neuromorphic computing based on carbon nanotubes. The collaboration between materials scientists, computer engineers, neuroscientists is highly required to demonstrate a stretchable soft machine [194] and a neuromorphic computer system [195].

Printable dielectrics such as ion gel may shed light on the fabrication of high-performance flexible carbon nanotube transistors [171]. Moreover, the flexible and stretchable electronics based on carbon nanotubes continue to amaze society and the community with more breakthroughs.

In summary, SWCNTs have demonstrated enormous excellence in electronics, biosensing, artificial intelligence, and the Internet of Things. Indeed, the understanding of the chirality-controlled synthesis of carbon nanotubes has pushed closer its applications to industrial mass production.
